# Leber Congenital Amaurosis Associated with *AIPL1*: Challenges in Ascribing Disease Causation, Clinical Findings, and Implications for Gene Therapy

**DOI:** 10.1371/journal.pone.0032330

**Published:** 2012-03-06

**Authors:** Mei Hong Tan, Donna S. Mackay, Jill Cowing, Hoai Viet Tran, Alexander J. Smith, Genevieve A. Wright, Arundhati Dev-Borman, Robert H. Henderson, Phillip Moradi, Isabelle Russell-Eggitt, Robert E. MacLaren, Anthony G. Robson, Michael E. Cheetham, Dorothy A. Thompson, Andrew R. Webster, Michel Michaelides, Robin R. Ali, Anthony T. Moore

**Affiliations:** 1 Department of Genetics, Institute of Ophthalmology, University College London, London, United Kingdom; 2 Moorfields Eye Hospital, London, United Kingdom; 3 Great Ormond Street Hospital for Children, London, United Kingdom; 4 Oxford University Hospitals NHS Trust, London, United Kingdom; University of Florida, United States of America

## Abstract

Leber Congenital Amaurosis (LCA) and Early Childhood Onset Severe Retinal Dystrophy are clinically and genetically heterogeneous retinal disorders characterised by visual impairment and nystagmus from birth or early infancy. We investigated the prevalence of sequence variants in *AIPL1* in a large cohort of such patients (n = 392) and probed the likelihood of disease-causation of the identified variants, subsequently undertaking a detailed assessment of the phenotype of patients with disease-causing mutations. Genomic DNA samples were screened for known variants in the *AIPL1* gene using a microarray LCA chip, with 153 of these cases then being directly sequenced. The assessment of disease-causation of identified *AIPL1* variants included segregation testing, assessing evolutionary conservation and *in silico* predictions of pathogenicity. The chip identified *AIPL1* variants in 12 patients. Sequencing of *AIPL1* in 153 patients and 96 controls found a total of 46 variants, with 29 being novel. *In silico* analysis suggested that only 6 of these variants are likely to be disease-causing, indicating a previously unrecognized high degree of polymorphism. Seven patients were identified with biallelic changes in *AIPL1* likely to be disease-causing. In the youngest subject, electroretinography revealed reduced cone photoreceptor function, but rod responses were within normal limits, with no measurable ERG in other patients. An increasing degree and extent of peripheral retinal pigmentation and degree of maculopathy was noted with increasing age in our series. *AIPL1* is significantly polymorphic in both controls and patients, thereby complicating the establishment of disease-causation of identified variants. Despite the associated phenotype being characterised by early-onset severe visual loss in our patient series, there was some evidence of a degree of retinal structural and functional preservation, which was most marked in the youngest patient in our cohort. This data suggests that there are patients who have a reasonable window of opportunity for gene therapy in childhood.

## Introduction

Leber Congenital Amaurosis (LCA) is an inherited retinal dystrophy characterised by visual impairment and nystagmus from birth or early infancy [1], [2]. It is clinically and genetically heterogeneous. There are currently 16 genes associated with LCA which account for approximately 70% of patients, and encode a variety of proteins, including those involved in developmental and physiological pathways in the retina [1], [2].

The safety and efficacy demonstrated by the on-going clinical trials of gene therapy for LCA associated with *RPE65* deficiency have provided proof-of-principle for other forms of LCA; thereby establishing a greater need for making timely and accurate molecular diagnoses [3]–[5]. It is also important to investigate the associated retinal phenotypes to help prioritise screening and to identify which genetic subtypes have a window of opportunity where photoreceptor rescue may be possible [1], [2], [6].

*AIPL1* (aryl hydrocarbon receptor-interacting protein-like 1) is expressed in rod and cone photoreceptors, and has a critical role in cell viability [7], [8]. It is required for the assembly of the phototransduction protein, phosphodiesterase, in both rods and cones [8]. Mutations in *AIPL1* are associated with a form of LCA that is characterised by maculopathy and a pigmentary retinopathy which is evident from a young age [9]–[13]. Retinal imaging studies have demonstrated significant loss of outer retinal structure at the central macula, with areas of relative preservation peripherally [9], [11], [13]. However, these findings principally relate to adult patients, and the finding that the presumed loss of photoreceptors on the basis of optical coherence tomography, is more severe with age, suggests that younger patients may have a greater degree of retained photoreceptors, which could be targeted by gene-supplementation therapy. Autofluorescence (AF) imaging undertaken in a recent study identified areas of retained AF at the posterior pole, which may be consistent with the presence of viable photoreceptors [9]. Importantly, retained rod function on dark-adapted perimetry and pupillometry has also been observed in patients, some of whom were in the third and fourth decades of life [11].

In mouse models of Aipl1 deficiency, rapid complete degeneration of both rods and cones is seen within 4 weeks of age [14], [15]. Significant rescue of photoreceptor structure and function has been achieved using adeno-associated viral vector-mediated gene supplementation in *Aipl^−/−^* mouse models of LCA following treatment of neonatal mice [9], [16], [17]. These studies suggest that a similar approach may be effective in humans. Although most patients have severe visual loss from infancy, a patient with a hypomorphic *AIPL1* genotype has been reported with a milder later-onset retinitis pigmentosa-like phenotype [11]. Such patients are more likely to respond to gene therapy and it may be worthwhile to screen *AIPL1* in patients with less severe forms of inherited retinal disease.

We have undertaken a study to identify patients with *AIPL1* mutations from our LCA and EOSRD cohort using a combination of microarray LCA chip analysis and direct sequencing, and thereby establish the prevalence of *AIPL1* sequence variants in our patient population [18], [19]. We have subsequently examined their phenotype in detail to gain insights into disease severity, variability and potential time-points that may be best to consider therapeutic intervention.

## Results

### Molecular Investigations

The Asper LCA microarray chip identified *AIPL1* sequence variants in 12 patients. Sequencing of *AIPL1* in 153 patients and 96 controls found a total of 46 variants (Table 1); with 29 being previously unreported (Table 1). No novel nonsense, frameshift or in/dels were found in the patient cohort.

**Table 1:** Variants identified in AIPL1 in patients and controls.

rs Number	Position	Nucleotide Change	Amino Acid Change	Allele Freq in patients (n = 153)	Allele freq in controls (n = 96)	HapMap CEU Allele Freq	AIPL1 disease allele
rs7211442	Intron 1	c.1-106C>A		C = 0.974, A = 0.026	C = 1.000, A = 0.000		
Novel	Intron 1	c.1-45C>A		C = 1.000, A = 0.000	C = 0.99, A = 0.01		
Novel	Exon 1	c.51G>A	p.Leu17Leu	G = 0.997, A = 0.003	G = 1.000, A = 0.000		
Novel	Intron 2	c.97-16C>T		C = 1.000, T = 0.000	C = 0.995, T = 0.005		
rs11650007	Exon 2	c.111C>T	p.Phe37Phe	C = 0.977, T = 0.023	C = 0.979, T = 0.021	C = 0.967, A = 0.033	
Novel	Exon 2	c.190G>A	p.Gly64Arg	G = 0.997, A = 0.003	G = 1.000, A = 0.000		Yes
Novel	Exon 2	c.264G>A	p.Trp88Ter	G = 0.987 A = 0.013	G = 1.000, A = 0.000		Yes
Novel	Exon 2	c.267C>T	p.Cys89Cys	C = 1.000, T = 0.000	C = 0.979, T = 0.021		
rs12449580	Exon 2	c.268G>C	p.Asp90His	G = 0.895, C = 0.105	G = 0.823, C = 0.177	G = 0.667, C = 0.333	
Novel	Intron 2	c.277-30insG		G = 0.997, GG = 0.003	G = 1.000, insG = 0.000		
rs12453262	Intron 2	c.277-10 A>G		A = 0.775, G = 0.225	A = 0.823, G = 0.177	A = 0.456, G = 0.544	
Novel	Intron 2	c.277-2A>G		A = 0.997, G = 0.003	A = 1.000, G = 0.000		Yes
rs62619924	Exon 3	c.286G>A	p.Val96Ile	G = 0.984, A = 0.016	G = 1.000, A = 0.000	G = 0.992, A = 0.008	
rs8075035	Exon 3	c.300A>G	p.Leu100Leu	A = 0.709, G = 0.291	A = 0.833, G = 0.167	A = 0.372, G = 0.372	
rs8069375	Exon 3	c.341C>T	p.Thr114Ile	C = 0.99, T = 0.01	C = 1.000, G = 0.000	C = 0.926, T = 0.074	
Novel	Exon 3	c.390C>A	p.His130Gln	C = 0.997, A = 0.003	C = 1.000, A = 0.000		
rs16955851	Exon 3	c.496A>T	p.Tyr134Phe	A = 0.999 T = 0.001	A = 1.000, T = 0.000	A = 1.000, T = 0.000†	
Novel	Exon 3	c.439C>T	p.Leu147Leu	C = 0.993, T = 0.007	C = 0.993, T = 0.007		
rs925615	Intron 3	c.466-26T>C		T = 0.938, C = 0.062	T = 0.906, C = 0.094	T = 0.903, C = 0.097	
Novel	Intron 3	c.466-2A>G		A = 0.993, G = 0.007	A = 1.000, G = 0.000		
rs62637009	Exon 4	c.487C>T	p.Gln163Ter	C = 0.993, T = 0.007	C = 1.000, T = 0.000	N/P	Yes
Novel	Exon 4	c.555A>G	p.Gly185Gly	A = 0.997, G = 0.003	A = 1.000, G = 0.000		
Novel	Exon 4	c.592T>A	p.Ser198Thr	T = 0.984, A = 0.016	T = 1.000, A = 0.000		
Novel	Exon 4	c.593C>T	p.Ser198Phe	C = 0.997, T = 0.003	C = 1.000, T = 0.000		
Novel	Exon 4	c.641A>G	p.Lys214Arg	A = 0.997, G = 0.003	A = 1.000, G = 0.000		
rs925616	Intron 4	c.642+48G>A		G = 0.791, A = 0.209	G = 1.000, A = 0.000	G = 0.625, A = 0.375	
rs2292545	Intron 4	c.642-33C>T		C = 0.863, T = 0.137	C = 0.964, T = 0.036	C = 0.717, T = 0.283	
rs2292546	Exon 5	c.651A>G	p.Pro217Pro	A = 0.614, G = 0.386	A = 0.724, G = 0.276	G = 0.717, A = 0.283	
Novel	Exon 5	c.678G>A	p.Glu226Glu	G = 1.000, A = 0.000	G = 0.974, A = 0.026		
rs62637013	Exon 5	c.784G>A	p.Gly262Ser	G = 0.997, A = 0.003	G = 1.000, A = 0.000	N/P	Yes
Novel	Intron 5	c.784+8G>C		G = 0.984, C = 0.016	G = 1.000, C = 0.000		
rs7222126	Intron 5	c.784+18G>A		G = 0.984, A = 0.016	G = 1.000, A = 0.000	N/P	
Novel	Intron 5	c.784+26G>C		G = 0.987, C = 0.013	G = 1.000, C = 0.000		
rs62637014	Exon 6	c.834G>A	p.Trp278Ter	G = 0.98, A = 0.02	G = 1.000, A = 0.000	N/P	Yes
Novel	Exon 6	c.894G>C	p.Gln298His	G = 0.997, C = 0.003	G = 1.000, C = 0.000		
rs62637015	Exon 6	c.905G>T	p.Arg302Leu	G = 0.967, T = 0.033	G = 1.000, T = 0.000	N/P	?
Novel	Exon 6	c.971G>T	p.Arg324Leu	G = 0.997, T = 0.003	G = 0.99, C = 0.01		
Novel	Exon 6	c.1005C>A	p.Pro335Pro	C = 1.000, A = 0.000	G = 0.99, C = 0.01		
Novel	Exon 6	c.1023G>A	p.Glu341Glu	G = 1.000, A = 0.000	G = 0.974, A = 0.026		
Novel	Exon 6	c.1032A>G	p.Ala344Ala	A = 0.99, G = 0.01	A = 1.000, G = 0.000		
Novel	Exon 6	c.1038A>G	p.Ser346Ser	A = 0.99, G = 0.01	A = 1.000, G = 0.000		
Novel	Exon 6	c.1091C>G	p.Ala364Gly	C = 0.997, G = 0.003	C = 1.000, G = 0.000		
Novel	Exon 6	c.1097C>G	p.Pro366Arg	C = 0.993, G = 0.007	C = 1.000, G = 0.000		
Novel	Exon 6	c.1110A>T	p.Pro370Pro	A = 1.000, T = 0.000	A = 0.995, T = 0.005		
rs61757484	Exon 6	c.1126C>T	p.Pro376Ser	C = 0.984, T = 0.016	C = 1.000, T = 0.000	C = 0.933, A = 0.067*	?
Novel	Exon 6	c.1164A>G	3′UTR	A = 0.997, G = 0.003	A = 1.000, G = 0.000		

* Allele frequency taken from AGI-ASP population on dbSNP. This population is from the Cornell apparently healthy population and the frequency is based on 60 chromosomes.

† The minor allele has a frequency in the African American population of 0.022.

Analysis of all variants for their effect on splicing is shown in supplementary table 1 Table S1). Human splicing factor matrix analysis showed that 22 variants showed the potential to affect splicing in *AIPL1*. Without further experimental analysis, it is difficult to decide which of these splicing effects would occur in vivo.

*In silico* mutation analysis programs were used to identify which of the 17 missense variants had the potential to be disease-causing (Table 2). Variants p.Asp90His, p.Val96Ile and p.Thr114Ile are known SNPs in *AIPL1* (Tables 1 and 2). The substitution p.Arg324Leu was identified in our control panel and predicted to not be disease-causing by two of the analysis programs (Tables 1 and 2) and showed no effect on splicing, and was therefore considered to be a SNP. Analysis of the variants, p.Tyr134Phe, p.Ser198Thr, p.Lys214Arg and p.Pro376Ser, considered them to be benign rare variants. Only p.Lys214Arg was predicted to potentially have an effect on splicing. A second mutation was not found in the patients harbouring these variants.

**Table 2:** In silico analysis of all missense variants identified in AIPL1 in this study.

Change	SIFT		Polyphen 2		pMUT			Consensus
	Prediction	Tolerance index	Prediction	Hum Varscore	NNoutput	Reliability	Prediction	
p.Gly64Arg	Intolerant	0.00	Probably damaging	1.000	0.6670	3	Pathological	Disease causing
p.Asp90His	Tolerant	0.26	Benign	0.241	0.4719	0	Neutral	SNP(rs12449580)
p.Val96Ile	Tolerant	0.20	Benign	0.064	0.0794	8	Neutral	SNP(rs62619924)
p.Thr114Ile	Tolerant	0.19	Benign	0.48	0.7499	4	Pathological	SNP (rs8069375)
p.His130Gln	Tolerant	0.71	Probably damaging	0.961	0.3446	3	Neutral	Uncertain
p.Tyr134Phe	Tolerant	0.26	Benign	0.448	0.0715	8	Neutral	Benign rare variant
p.Ser198Thr	Tolerant	0.60	Benign	0.002	0.2553	4	Neutral	Benign rare variant
p.Ser198Phe	Tolerant	0.07	Benign	0.072	0.6788	3	Pathological	Uncertain
p.Lys214Arg	Tolerant	0.70	Possibly damaging	0.688	0.0734	8	Neutral	Benign rare variant
p.Gly262Ser	Tolerant	0.86	Possibly damaging	0.608	0.3055	3	Neutral	Benign rare variant
p.Gln298His	Tolerant	0.06	Benign	0.174	0.5535	1	Pathological	Uncertain
p.Arg302Leu	Tolerant	0.16	Benign	0.003	0.8448	6	Pathological	Uncertain
p.Arg324Leu	Tolerant	0.20	Benign	0.204	0.8587	7	Pathological	Uncertain
p.Ala364Gly	Tolerant	0.48	N/D	N/D	0.1970	6	Neutral	Benign rare variant
p.Pro366Arg	Tolerant	0.10	N/D	N/D	0.2874	5	Pathological	Uncertain
p.Pro376Ser	Intolerant	0.00	N/D	N/D	0.2874	4	Neutral	Uncertain

N/D = Unable to make a prediction due to lack of data.

If all three programs agree that the change is pathological, then the consensus is disease causing. If one or two out of three programs agree with the variant being pathological, this is labelled uncertain. If all three agreed the change was benign (or in the case of polyphen 2 possibly damaging), then the variant is labelled a SNP (if rs number is available) or Benign rare variant.

The pathogenicity of the variants p.His130Gln, p.Ser198Phe, p.Gln298His and p.Pro366Arg was inconclusive with at least one of the three programs suggesting that these changes could be disease-causing. Only p.Gln298His showed the potential to break its nearest acceptor splice site. They were also not found in controls, nor listed on dbSNP. None of the patients with these changes had a second variant identified. Therefore we were unable to conclusively establish whether these variants are benign rare variants or disease-causing mutations.

We believe that two previously reported mutations in *AIPL1* that have been suggested to be disease-causing, are more likely to be benign variants. We identified two patients who were homozygous for p.Arg302Leu, with a further four patients found to be heterozygous for this change, with no second mutation identified (Table 1). This change has been previously published as a mutation [20]. This allele has no published allele frequency in white controls, but was detected in our laboratory in the homozygous state in an unaffected parent of Indian descent whose affected children have LCA caused by a homozygous mutation in the gene *RPGRIP1*. All our patients with this change were from the Indian subcontinent and the Middle-East, suggesting it may be a polymorphism in these populations. Another allele found in our patient cohort is p.Pro376Ser, published as a mutation in 2000 [20]. This allele is not found in white controls but has been found in African Americans at a low frequency of 0.022 (Table 1). Interestingly, the patients in our cohort with this change are of West African descent. We found one patient who was homozygous for the change. Her affected half sister was heterozygous for p.Pro376Ser, and we were unable to find a second variant. This family is now being investigated for other disease-causing genes. A third patient of Caribbean descent was also found to have this change in the heterozygous state with no second mutation. *In silico* analysis did not identify this variant as likely to be disease-causing (Table 2).

Using a combination of sequencing to confirm Asper chip variants plus complete sequencing of the entire coding region of *AIPL1*, this study has identified seven patients with biallelic changes in *AIPL1* that are highly likely to be disease-causing (Table 3). Interestingly, the microarray chip identified either one or both *AIPL1* variants in all 7 patients. Five patients were found to have homozygous null mutations, one patient was a compound heterozygote for a missense and a nonsense mutation, and one patient was a compound heterozygote for a missense and splice site mutation (Table 3). One novel change was identified (p.Gly64Arg), with the most frequent mutation identified being p.Trp278Ter.

**Table 3:** Patients with disease-causing mutations in *AIPL1*.

Patient	Allele 1	Allele 2	Diagnosis	Ethnicity	Consanguinity
1	p.Trp278Ter	p.Trp278Ter	LCA	African	No
2	p.Trp278Ter	p.Trp278Ter	LCA	White	No
3	p.Gln163Ter	p.Gln163Ter	LCA	Middle-East	Yes
4	p.Trp88Ter	p.Trp88Ter	LCA	Pakistani	Yes
5	p.Trp88Ter	pTrp88Ter	LCA	Pakistani	Yes
6	c.277-2A>G	p.Gly262Ser	LCA	White	No
7	pGly64Arg	p.Trp278Ter	LCA	White	No

### Clinical Assessment

Seven patients were identified harbouring two potential disease-causing alleles in *AIPL1*. Clinical findings are summarised in Table 4. All subjects presented at birth or early infancy (within 6 months) with nystagmus; with reduced vision also noted in six of these patients. The age at examination ranged from 2 years to 36 years, with two patients examined at an early age (2 years; cases 3 and 7). Photoattraction (light staring) was reported in two subjects.

**Table 4:** Summary of Clinical Characteristics of Patients with Disease Causing Mutations in *AIPL1*.

Case	Ethnicity	Mutation	Age at onset	Presenting symptoms	Age at examination (years)	BCVA LogMAR (OD; OS)	Nystagmus	Strabismus	Pupils	PAV/PAT	Pigmentary retinopathy	Maculopathy	Disc pallor/drusen	White retinal dots	Cataract/keratoconus	Refraction	Other features
1	African	p.Trp278Ter p.Trp278Ter	Birth	Poor vision, nystagmus	19	PL;PL	Y	N	Am	N	Moderate, bone spicules	Mild Mottling	N	Y	N	Moderate myopia (−5.50)	
2	White	p.Trp278Ter p.Trp278Ter	Birth	Poor vision, nystagmus	24	PL;PL	Y	N	ND	N	Moderate, bone spicules	Atrophy	N	N	NS	ND	
3	Arab	p.Gln163Ter p.Gln163Ter	2 months	Poor vision, nystagmus	2	PL;PL	Y	ET	Am	PAT	None	None	N	N	N	Moderate hyperopia (+6.50)	
4	Pakistani	p.Trp88Ter p.Trp88Ter	Birth	Poor vision, nystagmus	30	PL;PL	Y	XT	Am	N	Moderate	Atrophy	Y	N	PS, KC	ND	
5	Pakistani	p.Trp88Ter p.Trp88Ter	Birth	Poor vision, nystagmus	36	PL; PL	Y	XT	Am	N	Moderate	Atrophy	Y	N	PS, KC	ND	
6	White	c.277-2A>G p.Gly262Ser	6 months	Poor vision, nystagmus	17	PL;PL	Y	N	Am	PAT	Moderate	Atrophy	DR	Y	PS	Moderate myopia (−5.00)	
7	White	p.Gly64Arg p.Trp.278Ter	2.5 months	Nystagmus	2	0.5 BEO	Y	N	Normal	N	None	Mild Mottling	N	N	N	High myopia (−8.50)	Head nodding

BCVA – best corrected visual acuity; PL - perception of light; BEO – both eyes open; PL - perception of light; XT - exotropia; ET - esotropia; N – no; ND – not done; Am –amaurotic; NS - nuclear sclerosis; OD - right eye; OS - left eye; PAT- photoattraction; PAV - photoaversion; DR- drusen; PS - posterior subcapsular cataract; KC – keratoconus; Y – yes.

Only case 7 (2 years of age) with a BCVA of 0.5 LogMAR, had a visual acuity better than perception of light. Four subjects (aged 17 to 36 years) had evidence of cataract (3 had posterior subcapsular lens opacity, and 1 nuclear sclerosis), with 2 of these patients also having keratoconus (Table 4). Three patients had a myopic refractive error, whilst one had moderate hypermetropia.

**Figure 1:**
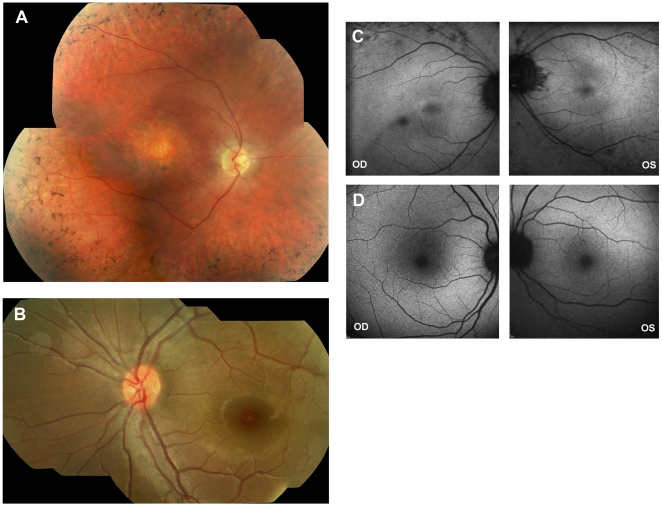
Colour fundus photographs and autofluorescence imaging of patients with AIPL1-associated retinal dystrophy. A. Colour fundus photograph of the right eye of case 2 at age 24 years, showing typical findings in an older subject with *AIPL1*-related retinal dystrophy. Characteristic features include bone spicule retinal pigmentation in the mid-periphery, macular atrophy and optic nerve pallor. B. Colour fundus photograph of the left eye of case 3 at age 2 years. The fundus appears unremarkable with minimal changes at the fovea. C. Autofluorescence (AF) imaging of case 1 at age 19 years showing evidence of a mild generalised reduction in AF at the posterior pole, with relatively preserved macular AF. D. Autofluorescence imaging in case 6 at age 17 showing good preservation of macular AF and some loss of foveal AF secondary to atrophy.

An increasing degree and extent of retinal pigmentation, including bone spicule formation, was noted with increasing age; with the two youngest subjects (cases 3 and 7) having no retinal pigmentation (Table 4; Figure 1). Two patients were found to have retinal white dots (cases 1 and 6). The severity of maculopathy also increased with age, with patients developing frank full-thickness retinal atrophy over time; again the two youngest subjects had a milder phenotype, with either a normal appearing macula or mild retinal pigment epithelial mottling (Table 4; Figure 1). Optic nerve pallor was noted in the two oldest subjects in our series, with one patient (case 6) having disc drusen.

Autofluorescence images were obtained in 2 patients (Figure 1). In case 1 there was evidence of a mild generalised reduction in AF at the posterior pole, with relatively preserved macular AF. Reduced foveal AF was observed, corresponding to the atrophy seen clinically in case 6, with otherwise relatively well preserved macular AF.

**Figure 2:**
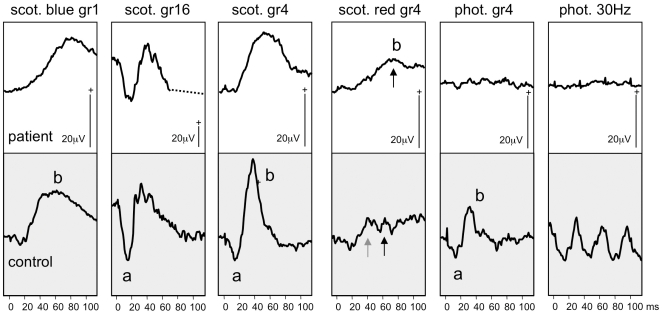
Scotopic and photopic ERG recordings of patient case 7. ERGs recorded from case 7 aged 14 months, using skin electrodes to a range of flash strengths (Grass (gr) 1–16) presented scotopically and photopically are shown above age-matched control data in the lower grey panel. These ERGs indicate cone photoreceptor dysfunction; evidenced by markedly reduced photopic cone and 30 Hz flicker ERGs, and a scotopic red flash ERG that shows predominance of the later rod dominated b-wave (arrowed). The scotopic (gr4) mixed rod cone waveform lacks an a-wave and the time to peak is increased. In contrast predominantly rod mediated function is within the normal range; evidenced by the normal a-wave to a maximal scotopic flash and rod driven b-wave to a scotopic dim blue flash.

Electrophysiological assessment was undertaken in 6 patients and revealed no measurable rod or cone responses in five subjects (cases 2 to 6). It is of note that in one of the youngest subjects (case 7), rod responses were within normal limits, with recordable but significantly reduced cone photoreceptor function (Figure 2).

## Discussion

We have identified seven patients who harbour biallelic mutations in *AIPL1* that are highly likely to be disease-causing. The detailed molecular screening we have undertaken in a large panel of patients recruited from a single institution has demonstrated the previously unrecognised high degree of polymorphism present in *AIPL1*, which complicates the interpretation of any sequence variants that are found. This may explain the difference between the previously reported prevalence of *AIPL1*-associated disease (approximately 7%), and that observed in our study (approximately 2%) [20]. We have carefully probed the likely disease association of each of the variants in order to establish as reliably as possible a molecular diagnosis. This is critical if there is to be any gene-specific therapeutic interventions. In keeping with previous studies, we have identified the p.Trp278Ter as the most common disease variant, and also shown that null alleles are the most prevalent disease-associated variants [9]–[11], [20].

Direct sequencing of a large number of patients in a condition that is known to be caused by at least 16 genes is a time consuming and expensive procedure. The use of APEX chips such as the Asper LCA chip can be an effective first screen allowing patients with known mutations to be identified quickly and then removed from the cohort for any further screening. In this study we screened 392 individuals using the LCA APEX chip, with one or more alleles in an LCA gene identified in approximately 40% of these patients. We elected to screen 153 of the remaining patients who either had 1 or more alleles in *AIPL1* or had no alleles identified in other genes. This number was based on human, financial and time constraints. It is plausible that by not sequencing the remaining 239 DNAs, we may have missed some *AIPL1* positive patients. Direct sequencing of the 306 alleles (153 patients) only identified 1 variant that was considered to have the potential to be disease-causing; a frequency of detection for *AIPL1* alleles of 0.33%. On this basis, sequencing the remaining 239 DNAs could be predicted to identify 0.78 alleles, which would not have increased the number of patients identified in this study. Newer techniques, such as next generation sequencing (NGS) of either the whole genome or exome, will allow a higher rate of mutation detection, and as the cost decreases NGS will be used more commonly

This large screen only identified one potential novel change, p.Gly64Arg in patient 7 (Table 3). This variant was not found in the other 152 patients we screened, nor in the 96 ethnically matched controls. *In silico* analysis suggests that it has the potential to be disease-causing (Table 3). We have included the variant p.Gly262Ser, present in patient 6, as a disease-causing mutation. It has been previously published as a disease-causing allele [20]. *In silico* analysis shows that only 1 out of the 3 programs suggests that it is disease-causing (Table 2). This variant may have an effect on splicing as shown in supplementary table 1. It is paired with the published mutation c.277-2A>G^28^. Both of these changes were previously published in a heterozygous state with the known nonsense mutation p.Trp278Ter [20]. Expression of p.Gly262Ser has suggested that the mutated protein will still bind to NUB1 [21], but may increase its affinity compared to the wildtype protein [22]. This variant does not affect the ability of AIPL1 to interact with Hsp90 and Hsp70 [23].

We found at least 4 novel variants (p.His130Gln, p.Ser198Phe, p.Gln298His and p.Pro366Arg) for which *in silico* analysis proved inconclusive. We were also not able to find a second disease-causing variant in these patients. This may either indicate that these variants are benign and rare in the population and not disease-causing, or that these patients have a second disease mutation which was missed by Sanger sequencing of the coding regions of *AIPL1*.

Two previously reported disease-associated variants are more likely to represent rare ethnic SNPs. We believe that our patients provide evidence that p.Arg302Leu and p.Pro376Ser are not *AIPL1* mutations. For p.Arg302Leu we identified two brothers who were heterozygous for the change but homozygous for a mutation in *RPGRIP1*. Further examination of the family showed no second mutation and that their unaffected father was homozygous for p.Arg302Leu. This change has been previously published as a mutation but has only been seen in patients of Indian/Middle East descent. Further evidence supporting a lack of pathogenicity of the p.Arg302Leu variant includes that this amino acid residue is not conserved across species, in expression analysis it does not affect the way AIPL1 interacts with NUB1 [21], [22], Hsp70 or Hsp90 [23], and also does not affect the ability of AIPL1 to interact with farnesylated proteins [24]. We also propose that p.Pro376Ser, previously published as a mutation is a rare SNP in the African population. dbSNP has a frequency for this SNP as 0.022 in the African American population. All three patients in our cohort were of West African descent. *In silico* analysis did not identify this variant as likely to be disease-causing (Table 2) and *in vitro* expression of the mutant protein has shown that it will still interact with NUB1 [21].

All seven subjects presented at birth or early infancy (within 6 months) with nystagmus; with reduced vision also noted in six of these patients. Consistent with previous reports, photoattraction was described in two subjects; this is not a specific finding as it has been described in other LCA/EOSRD genotypes [1], [2], [9]. Although refractive data was available in only 4 subjects, it is interesting that 3 of these had a moderate to high myopic refractive error, in contrast to the predominant hypermetropia recently reported [9], [11]. Only one patient had a BCVA better than perception of light, with 0.5 LogMAR recorded at 2 years of age. In keeping with previous studies, cataract (60% patients) and keratoconus (30%) was common in our cohort [9], [10], [20].

An increasing degree and extent of retinal pigmentation, including bone spicule formation, was noted with increasing age, with the two youngest subjects not having any evidence of retinal pigmentation. The severity of maculopathy also increased with age, with patients developing marked atrophy over time. The two youngest subjects had a milder phenotype, either a normal appearing macula or mild retinal pigment epithelial mottling. These findings suggest that at the early stages of disease there may be a relative degree of peripheral and central preservation of retinal structure, with clear implications for therapeutic interventions. Optical coherence tomography (OCT) in infancy, using hand-held probes, would be valuable to investigate this further. In older subjects there is OCT evidence of reduced retinal thickness and/or integrity of retinal lamination in the presence of relatively normal ophthalmoscopic findings; although parafoveal retinal structure has been shown to be preserved in some patients [9], [11]. However, patients with *AIPL1* disease have yet to be identified and studied in infancy with high-resolution quantitative retinal imaging. Adaptive optics imaging, which is directly complementary to OCT, is likely to be helpful in establishing *in vivo* whether target photoreceptors are present and to what extent and at what retinal locations.

In keeping with the clinical examination findings suggestive of a degree of retinal architecture preservation in younger patients, autofluorescence (AF) imaging, whilst showing evidence of generalised reduction in AF at the posterior pole, also demonstrated relatively preserved macular AF; suggestive of outer segment turnover and thereby a degree of structurally intact photoreceptors in complex with retinal pigment epithelial cells. Interestingly, the retained AF signal that was observed in the two imaged patients had a BCVA of perception of light. These observations are in agreement with those reported by Testa et al recently [9].

No measurable rod or cone ERG responses were observed in five subjects, in keeping with previous reports [9]–[11]. It is of note that we have identified a patient (2 years old) with rod responses within normal limits, with recordable but significantly reduced cone photoreceptor function. This important finding of preserved retinal function has also recently been reported in a case series of 3 young patients with *AIPL1*-associated disease [25].

Most reported cases of *AIPL1*-associated disease have an early onset severe retinal dystrophy, which poses challenges with regard to potential treatment. The significant rescue demonstrated in animal models with severe rapidly progressive disease by several independent studies is supportive of a potential therapeutic effect in patients [9], [16], [17]. The severe phenotype seen in most older patients suggests that therapy will need to be given in early childhood. Our identification of a patient who at a young age (2 years) has evidence of a milder phenotype, including normal retinal examination, useful central vision, normal pupil responses, and relatively preserved electrophysiological function (rod to a greater extent than cone), suggests that there are patients who have a reasonable window of opportunity for therapy in childhood. It is of note that this patient harbours a novel missense mutation rather than the more common homozygous null genotype, and this potentially hypomorphic allele may underlie her milder phenotype. It is possible that patients with a milder phenotype have not been identified as only patients with severe disease have been screened. There does appear to be a subgroup of patients with less severe later onset disease who may also be amenable to therapy, suggesting that later onset cohorts should also be screened for *AIPL1* mutations [11].

Identifying patients shortly after birth will clearly be challenging, although the advent of next generation sequencing is likely to lead to the early identification of patients with a wider range of phenotypes (screening of exons of all genes known to cause retinal disease in patients will become more common than screening directed by patient phenotype). Earlier treatment whilst likely to be more efficacious from the point of view of better retained retinal structure and a lesser degree of established amblyopia, will be complicated by the inherent difficulty of reliably identifying treatment responses in pre-verbal children; an area of increasing research importance.

In summary, we have demonstrated the polymorphic nature of *AIPL1* following analysis of a large panel of patients and control subjects, and thereby highlighted the difficulty of reliably ascribing disease causation to identified variants. The molecular characterisation of LCA is important for information on prognosis, genetic counseling and also with the advent of gene therapy trials, for identification of suitable candidates for potential therapy. Despite the severe *AIPL1*-associated visual loss in our patient series, there was some evidence of a degree of retinal structural and functional preservation, which was most marked in the youngest patient in our cohort, suggesting that there are patients who may be good candidates for gene therapy in childhood.

## Methods

All patients in this study had a clinical diagnosis of LCA or EOSRD, with onset before 6 years of age. All provided informed written consent as part of a research project approved by Moorfields Eye Hospital Ethics Committee, and all investigations were conducted in accordance with the principles of the Declaration of Helsinki. Written informed consent was obtained from the next of kin, carers or guardians on the behalf of the children participating in this study.

### Molecular Investigations

Blood samples were collected and DNA extracted using the Puregene blood extraction kit (Invitrogen, Paisley, UK) following manufacturer's instructions. The patient panel consisted of 392 unrelated probands; 26% had parents who were related. Seventy percent of the DNA samples were from white subjects, of either British or other European backgrounds; 21% were of Asian extraction, mostly Pakistani or Indian; 3% were of Middle-Eastern origin; 1.6% were African; 0.6% were Chinese and the remainder were of mixed backgrounds. The control panel consisted of 96 DNA samples originating from a control population of randomly selected, non-related white UK blood donors (ECACC Human Random Control-1 DNA panel).

#### APEX Chip

A genomic DNA sample from 392 unrelated affected patients was sent to Asper Ophthalmics Ltd for analysis as described previously [18], [19]. 112 patients were analysed using the current LCA microarray test, that contains 641 disease-associated sequence variants identified in 13 LCA or early-onset retinitis pigmentosa genes: *AIPL1*, *CRB1*, *CRX*, *GUCY2D*, *LRAT*, *TULP1*, *MERTK*, *CEP290*, *RDH12*, *RPGRIP1*, *LCA5*, *RPE65*, and *SPATA7*. Four earlier versions of the LCA chip were also used during the course of the study; 134 patients using a chip containing 8 genes and 345 variants, 58 patients analysed for 10 genes and 435 variants, 16 patients analysed for 11 genes and 451 variants and 72 patients analysed for 12 genes and 493 variants. Samples in which mutations were identified in other LCA genes were excluded from further study. The majority of this work has been published elsewhere [26]–[30].

#### Mutation screening

A total of 153 patient DNA samples (153/392) then underwent bi-directional sequencing of all 6 exons of the *AIPL1* gene (NM_014336.3), including splice-site junctions. These 153 subjects were selected on the basis of either no mutations identified on the microarray chip or that they had been found to harbour one or more *AIPL1* sequence variants. Primer sequences are available upon request. All standard polymerase chain reactions (PCR) were performed in a total volume of 30 µl containing 200 µM dNTPs (Bioline London, UK), 20 µM of each primer, 1× reaction buffer, 1.5 mM MgCl_2_ (Bioline), 1 unit of Biotaq (Bioline), and 100 ng of DNA. PCR was carried out on a PTC200 DNA engine thermal cycler (Bio-Rad, Hemel Hempstead, UK). Cycling conditions were as follows: 2 minutes denaturation at 94°C, followed by 35 cycles of 94°C for 30 seconds, annealing temperature for 30 seconds and extension at 72°C for 30 seconds. A final extension of 72°C for 5 minutes completed the cycling conditions.

PCR products were visualised on a 2% agarose gel containing 0.05% ethidium bromide. The products were cleaned using multiscreen PCR filter plates (Catalogue no LSKMPCR10, Millipore, Watford, UK) prior to sequencing. PCR products were sequenced directly using the ABI Prism Big Dye terminator Kit (V3.1) in a 10 µl reaction. Samples were purified using the Montage cleanup kit (catalogue no LSK509624, Millipore) prior to being run on an ABI applied biosystems 3730 DNA sequencer (Applied Biosystems, Foster City, CA, USA).

Electropherograms were analysed for sequence changes using DNAStar computational software (DNAStar, Inc., USA). Sequencing data obtained from PCR products were analysed using SeqMan, a programme designed to detect potential alterations in the sequence. Any sequence changes identified were checked visually. When family samples were available segregation of potentially disease-causing variants was investigated. Missense mutations were analysed using 3 software prediction programs: SIFT (Sorting Intolerant from Tolerance (http://sift.jcvi.org/) [31], PolyPhen2 (http://genetics.bwh.harvard.edu/pph/index.html) [32] and pMUT (http://mmb.pcb.ub.es/PMut/) [33]. All variants were also analysed for their effect on splicing using the Human Splicing finder programme version 2.4.1 (http://www.umd.be/HSF/)

### Clinical Assessment

Patients harbouring variants that were likely to be disease-causing underwent clinical testing. Clinical evaluation included best-corrected visual acuity (BCVA) and dilated fundus examination. Colour fundus photography and fundus autofluorescence (AF) imaging using a confocal scanning laser ophthalmoscope (Heidelberg Retina Angiogram, HRA 2; Heidelberg Engineering, Heidelberg, Germany) was undertaken.

Electrophysiological assessment was performed, including a full-field electroretinogram (ERG) and pattern ERG (PERG), incorporating the protocols recommended by the International Society for Clinical Electrophysiology of Vision (ISCEV); or using a modified paediatric ERG protocol with skin electrodes, as previously described [34]–[38].

## Supporting Information

Table S1 **Analysis of allelic variants for their effect on splicing.** Analysis of 46 variants identified in *AIPL1* using the Human splicing finder version 2.4.1 reporting the results from the HSF matrix. The values for the wild type and mutant sequences are showed. The larger the difference between the values the greater that change that the variant can affect the splice site.
(DOC)
