# Supplementary material for: Leber Congenital Amaurosis Associated with AIPL1: Challenges in Ascribing Disease Causation, Clinical Findings, and Implications for Gene Therapy
Source: PLoS One. 2012 Mar 6;7(3):e32330. doi: 10.1371/journal.pone.0032330 (PMC3295755; doi:10.1371/journal.pone.0032330)
Supplement: Table S1 — Analysis of allelic variants for their effect on splicing. Analysis of 46 variants identified in AIPL1 using the Human splicing finder version 2.4.1 reporting the results from the HSF matrix. The values for the wild type and mutant sequences are showed. The larger the difference between the values the greater that change that the variant can affect the splice site. (DOC) [file pone.0032330.s001.doc]

**Supporting Information.**

**Table S1.**

| rs Number | Position | Nucleotide Change | Amino Acid Change | Site affected | HSF matrix results | | | |
| --- | --- | --- | --- | --- | --- | --- | --- | --- |
|  |  |  |  |  | Wild Type | Mutant | If cryptic site, exon length variation | Variation (%) |
| rs7211442 | Intron 1 | c.1-106C >A |  |  |  |  |  | N/A |
| Novel | Intron 1 | c.1-45C>A |  |  |  |  |  | N/A |
| Novel | Exon 1 | c.51G>A | p.Leu17Leu |  |  |  |  | No change |
| Novel | Intron 2 | c.97-16C>T |  |  |  |  |  | No Change |
| rs11650007 | Exon 2 | c.111C>T | p.Phe37Phe |  |  |  |  | No Change |
| Novel | Exon 2 | c.190G>A | p.Gly64Arg | Acceptor | 53.56 | 82.5 | -95 | New site (+54.05) |
| Novel | Exon 2 | c.264G>A | p.Trp88Ter | Donor | 82.95 | 56.12 | -13 | Site broken (-32.35) |
| Novel | Exon 2 | c.267C>T | p.Cys89Cys | Donor | 40.17 | 67.01 | -11 | New site (+66.8) |
| rs12449580 | Exon 2 | c.268G>C | p.Asp90His | Donor | 82.95 | 70.94 | -13 | -14.48 |
| Novel | Intron 2 | c.277-30insG |  |  |  |  |  | No change |
| rs12453262 | Intron 2 | c.277-10 A>G |  | Donor | 56.26 | 68.43 | NA | New site (+21.62) |
| Novel | Intron 2 | c.277-2A>G |  | Acceptor | 90.43 | 61.48 | 0 | WT site broken (-32.01) |
| rs62619924 | Exon 3 | c.286G>A | p.Val96Ile | Donor | 65.22 | 38.38 | -180 | Site broken (-41.15) |
| rs8075035 | Exon 3 | c.300A>G | p.Leu100Leu | Acceptor | 76.25 | 76.37 | -32 | +0.16 |
| rs8069375 | Exon 3 | c.341C>T | p.Thr114Ile | Acceptor | 81.52 | 73.83 | -67 | -9.42 |
| Novel | Exon 3 | c.390C>A | p.His130Gln |  |  |  |  | No change |
| rs16955851 | Exon 3 | c.496A>T | p.Tyr134Phe | Acceptor | 67.03 | 65.86 | -30 | -1.74 |
| Novel | Exon 3 | c.439C>T | p.Leu147Leu | Donor | 69.1 | 67.12 | -24 | -2.87 |
| rs925615 | Intron 3 | c.466-26T>C |  |  |  |  |  | No change |
| Novel | Intron 3 | c.466-2A>G |  | Acceptor | 85.77 | 56.82 | 0 | WT site broken (-33.75) |
| rs62637009 | Exon 4 | c.487C>T | p.Gln163Ter | Acceptor | 78.93 | 71.25 | -24 | -9.73 |
| Novel | Exon 4 | c.555A>G | p.Gly185Gly |  |  |  |  | No change |
| Novel | Exon 4 | c.592T>A | p.Ser198Thr | Acceptor | 77.86 | 74.64 | -135 | -4.13 |
| Novel | Exon 4 | c.593C>T | p.Ser198Phe | Acceptor | 77.86 | 77.25 | -135 | -0.79 |
| Novel | Exon 4 | c.641A>G | p.Lys214Arg | Acceptor | 54.64 | 83.58 | -176 | New site (+52.98) |
|  |  |  |  |  | 75.56 | 46.61 | NA | Site broken (-38.31) |
| rs925616 | Intron 4 | c.642+48G>A |  | Acceptor | 79.22 | 79.11 | NA | -0.15 |
| rs2292545 | Intron 4 | c.642-33C>T |  |  |  |  |  | No change |
| rs2292546 | Exon 5 | c.651A>G | p.Pro217Pro | Donor | 43.81 | 70.64 | -134 | New site (+61.26) |
| Novel | Exon 5 | c.678G>A | p.Glu226Glu | Acceptor | 72.41 | 43.46 | -36 | Site broken (-39.98) |
| rs62637013 | Exon 5 | c.784G>A | p.Gly262Ser | Acceptor | 84.94 | 56 | NA | Site broken (-34.08) |
|  |  |  |  | Acceptor | 43.91 | 72.86 | NA | New site (+65.92) |
|  |  |  |  | Donor | 87.86 | 77.28 | 0 | WT site broken (-12.04) |
| Novel | Intron 5 | c.784+8G>C |  | Acceptor | 82.43 | 84.34 | NA | +2.32 |
| rs7222126 | Intron 5 | c.784+18G>A |  | Acceptor | 37.5 | 66.45 | NA | New site (+77.19) |
| Novel | Intron 5 | c.784+26G>C |  | Acceptor | 71.71 | 42.77 | NA | Site broken (-40.36) |
|  |  |  |  | Donor | 80.63 | 53.79 | +25 | Site broken (-33.28) |
| rs62637014 | Exon 6 | c.834G>A | p.Trp278Ter | Donor | 58.52 | 66.86 | -325 | New site (+14.25) |
| Novel | Exon 6 | c.894G>C | p.Gln298His | Acceptor | 85.88 | 56.93 | -110 | Site broken (-33.71) |
| rs62637015 | Exon 6 | c.905G>T | p.Arg302Leu | Donor | 81.35 | 69.04 | -255 | -15.14 |
| Novel | Exon 6 | c.971G>T | p.Arg324Leu |  |  |  |  | No change |
| Novel | Exon 6 | c.1005C>A | p.Pro335Pro | Acceptor | 54.75 | 83.69 | -222 | New site (+52.87) |
| Novel | Exon 6 | c.1023G>A | p.Glu341Glu | Acceptor | 72.35 | 43.41 | -239 | Site broken (-40.01) |
| Novel | Exon 6 | c.1032A>G | p.Ala344Ala | Acceptor | 82.45 | 82.38 | -251 | -0.08 |
| Novel | Exon 6 | c.1038A>G | p.Ser346Ser | Acceptor | 74.79 | 74.91 | -263 | +0.15 |
| Novel | Exon 6 | c.1091C>G | p.Ala364Gly | Acceptor | 87.21 | 76.91 | -309 | -11.81 |
| Novel | Exon 6 | c.1097C>G | p.Pro366Arg | Acceptor | 74.35 | 72.54 | -323 | -2.44 |
| Novel | Exon 6 | c.1110A>T | p.Pro370Pro | Acceptor | 77.21 | 48.26 | -327 | Site broken (-37.49) |
| rs61757484 | Exon 6 | c.1126C>T | p.Pro376Ser | Acceptor | 90.55 | 91.26 | -351 | +0.78 |
| Novel | Exon 6 | c.1164A>G | 3’UTR |  |  |  |  | N/A |
